# Psychological well-being and job performance of nurses and midwives amidst COVID-19 in Ghana; a multi-group analysis

**DOI:** 10.1371/journal.pone.0303855

**Published:** 2024-08-28

**Authors:** Felix Kwame Opoku, Nester Kumiwaa Owusu

**Affiliations:** Department of Human Resource Management, School of Business, University of Cape Coast, Cape Coast, Ghana; University of Oulu: Oulun Yliopisto, FINLAND

## Abstract

The purpose of this study was to examine the effect of psychological well-being on job performance among nurses and midwives in rural and urban hospitals in Ghana amidst COVID-19. The study adopted a purely quantitative approach, using the explanatory research design. Data were collected from 262 nurses and midwives in two selected hospitals in the Central Region of Ghana. The Structural Equation Modelling was used to analyze the data collected. The results revealed that the levels of psychological well-being and job performance were high in both hospitals amidst the pandemic. Further, it was observed that although psychological well- being had significant influence on job performance among the nurses and midwives in both rural and urban communities, the effect was more significant in the urban settlements. Given these findings, the study recommended that in order to effectively improve the job performance of nurses and midwives, management must adopt policies such as, flexible work arrangements, leave policy, and counselling services to support their psychological well-being.

## Introduction

The devastation of COVID-19 pandemic was experienced by millions of people globally [1, 2]. The novelty of this pandemic, coupled with the absence of an appropriate cure, prompted several governments to institute control measures to reduce its spread. These measures became necessary because COVID-19 had an adverse effect on virtually every aspect of human life, reducing the psychological ability of mankind to envision any positive future life or business [1, 3]. It also caused adverse psychological emotions such as, anxiety, apprehension, feelings of helplessness and hopelessness, social distance, fear of contracting the coronavirus, the loss of fundamental sense of security, worries about financial pressure and depression [4, 5]. These conditions, according to previous studies [1, 5, 6] negatively affect employee performance. In order words, previous studies indicate that there is significant, positive correlation between psychological well-being and employee performance [4, 7, 8]. It is therefore, logical to reason that the neglect or non-satisfaction of employee psychological needs can lead to a decline in their job performance. Perhaps this was the reason why key performance indicators of both urban and rural hospitals in Ghana dropped at the initial stages of COVID-19 [9, 10].

Following the severity of the pandemic, the government of Ghana instituted several control measures such as, lockdowns, wearing of nose masks, ban on social and community events, strict hand washing protocols, school and company closures, travel restrictions, feeding allowances to the needy, and social distancing [5]. Although the financial and physical interventions saw significant improvements in slowing down the spread of the virus, these measures themselves could not subdue the psychological effect of the pandemic on health workers who, generally, are the first point of contact to patients who visit the health facilities [11]. In course of admitting patients, attending to emergencies or casualties, administering drugs and performing laboratory tests, the health workers get more exposed to the conditions of attracting the deadly coronavirus, thereby making them more vulnerable to the emotional and psychological effects of COVID-19 infection.

Much as the effect of COVID-19 pandemic is expected to affect the psychological well-being of health workers generally, previous studies [1, 12–14] have shown that the psychological well-being of rural health workers; following their infrastructural and materialistic disadvantage, is relatively hampered than their urban counterparts. However [15] found that the psychological well-being of rural workers is not solely affected by materialistic matters but also a number of non-material characteristics, meaning that the infrastructural and materialistic disadvantage of rural health workers alone cannot completely determine their psychological well-being. These conflicting findings imply that empirical data on the geographic pattern of employee psychological well-being is mixed and inconclusive with respect to health workers in both developed and developing countries [16]. Consistent with these findings, predicting the contextual variation of the performance of rural and urban health workers (nurses and midwives) in relation to their psychological well-being has become very crucial and critical, especially amidst COVID-19 pandemic. Against this background, the current study is conducted to examine the effect of psychological well-being on the job performance of health workers generally in Ghana, and to determine whether rurality can lead to differences in their psychological well-being and job performance, amidst COVID-19 pandemic.

## Literature review and development of hypotheses

### Theoretical issues underlying the study

#### Job-demands resource theory

The Job Demands-Resources (JD-R) theory is the main foundation on which this study is built. The theory was proposed by [17]. The JD-R theory posits that every job has a set of characteristics which can be classified into two general categories—job demands and job resources. Job demands are the psychological, physical, social, and other organizational aspects of the job that require physical, cognitive and emotional effort or skill from the incumbent. They are the features of the job that require energy, such as workload, demanding tasks, problems with equipment, long working hours, time pressure and disputes [18]. Job resources on the other hand, are the elements of the job that assist the incumbent to accomplish job expectations. They include performance reviews, social support, decent working conditions, and possibilities for progression [19]. Job resources are motivating factors that offer meaning to employees and help them to meet their core psychological requirements. The central assumption of the theory is that stress is a response to the imbalance between job demands and job resources [18]. When job demands are higher relative to accompanying job resources, stress, strain and emotional exhaustion develop and vice versa [19]. The theory also holds that although job demands are not necessarily negative, they may turn into job stressors when meeting those demands require high effort from which the employee fails to recover adequately. As the interplay between job demands and job resources exists in every job, the JD-R theory is applicable to all organizational settings, irrespective of the particular job demands and job resources involved [18]. The theory categorizes employees into two–burn-out employees and engaged employees. According to [18], whereas burned-out employees usually scramble for more job demands over time, engaged employees generally mobilize job resources to remain engaged.

### Conceptual issues underlying the study

#### Psychological well-being

[20] defined psychological well-being as an individual’s ability to maintain a positive state of mental and emotional health and operate in a mentally stable and functional manner. Psychological well-being is also defined by [21] as the state of happiness, based on the individual’s subjective experiences. Typical examples of psychological well-being include constructive interactions with colleagues, self-mastery, independence, a sense of drive and significance in life, autonomy, and feelings of purpose [22, 23]. [21] identified two forms of psychological well-being: hedonic component and eudaimonic component. [24] defined hedonic well-being as pleasure or affective experience that is characterized by the presence of positive emotions, life satisfaction, and the absence of negative emotions. Thus, the hedonic component consists of pleasurable experiences and pain avoidance. The eudaimonic component on the other hand, refers to quality of life enjoyed by a person as a result of growth and development of that person’s best potentials, and the application of that potential in the fulfillment of their personally expressive and self-concordant goals [21]. The effectiveness of a person’s total psychological functioning depends on both hedonic and eudaimonic components, although previous studies [25–28] have placed emphasis on the hedonic component than the eudaimonic component. For example [29, 30], argue that unhappy employees are more prone to having low self-esteem and reduced motivation (hedonic) at work, without considering the employee’s desire for a more fulfilled life (eudaimonic).

Psychological well-being generally relates to the quality of life of a person and the ability to live a life devoid of fear, anxiety or any form of mental strain. Although the concept is often used synonymously with happiness, it is evident from the preceding review that happiness is just a subset of psychological well-being. For the purpose of this study, psychological well-being is defined in line with the definition by [31] who describe it as a positive state of social, physiological, and mental condition. By implication, psychological well-being in the context of this study is not limited to the mere absence of sickness or sadness but the complete state of physical, emotional, social and mental wellness of the person.

#### Conceptualizing job performance

[32] defined job performance as “those observable behaviours under the control of the individual that contribute to the organization’s goals, and can be measured according to the individual’s level of proficiency”. Job performance is also defined by [33] as “the total expected value to the organization of the discrete behavioral episodes that an individual carries out over a standard period of time”. These definitions portray job performance as an input-output process where employees exert efforts to yield certain outcomes either positive or negative. As noted by [34], job performance can be seen from two angles; job relevant behaviors and work outcomes. Whereas job relevant behaviors encompass the efforts employees put in their jobs, work outcomes reflect the quality and quantity of work done. The concept of job performance is an important part in human resource management [35, 36]. Most of what human resource managers do gears towards having a positive impact on individual performance. Consequently, many organizations have spent considerable time and effort in attracting, retaining and motivating a highly performing set of individuals whose collective effort is capable of helping management to meet organizational goals such as delivering the goods and services consistent with the needs and expectations of customers; and achieving competitive advantage in the face of global competition [32, 33, 37].

There are no universally accepted number of dimensions of job performance [38, 39]. [40] original classification of the dimensions of job performance into task and contextual performance [41], added counterproductive work behaviour to the framework, resulting in a three-dimensional framework of job performance. [42] disregarded the counterproductive work behaviour dimension and replaced it with adaptive job performance dimension while [43] included all four dimensions in their framework.

#### Psychological well-being and employee performance

Previous studies [44–48] have shown that psychological well-being has significant positive association with employee performance. [47] examined the effect of psychological well-being on employee job performance in projectized and non-projectized organizations. The objectives of the study were two-fold: (a) to examine the psychological well-being of employees and its relationship with their levels of job performance, and (b) to compare psychological well- being of employees in projectized and non-projectized organizations. The authors chose 17 Information Technology Companies for the study. A total of 84 questionnaires were retried from respondents. The results revealed that high psychological well-being leads to increased employee performance in both projectized and non-projectized firms.

Much like [46, 47] researched into the happy worker-productive worker thesis, which suggests that individuals and groups with high well-being perform better in their jobs than do those with lower well-being. The study was mainly qualitative, looking into various forms of context-free and job-related well-being. The study also explored job performance in respect of in-role and extra-role behaviors through specific activities like creativity and proactiveness. The results showed significant links between well-being and employee job performance. The study also found that factors such as, autonomy, job rank and expected benefits significantly moderate the influence of individual well-being on employee job performance.

[44] also investigated the role of affective commitment as a mediator between psychological well-being and job performance. The study also considered the moderating role of job insecurity on the link between psychological well-being and affective commitment. Data were gathered from employees from cellular companies using paper-and-pencil surveys. A total of 280 responses were received. Hypotheses were tested using structural equation modeling and Hayes’s Model. Affective commitment was found to mediate the direct relationship between psychological well-being (hedonic and eudaimonic) and employee job performance. The findings from their study also suggest that job insecurity moderates the link between psychological well-being (hedonic and eudaimonic) and employee job performance. The study extended the current literature on employee well-being in two ways. First, it examined psychological well-being in terms of hedonic and eudaimonic well- being with employee work-related attitudes and behaviors. Second, it examined the significant role of perceived job insecurity between psychological well-being and affective commitment.

[49] examined the relationship between fear of COVID-19, psychological distress and coping (performance) strategies among undergraduate students in Ghana. The study employed a sample of 209 students. The results indicated normal to mild levels of psychological distress and above average scores on fear of coronavirus. The findings also emphasized the need to design and optimize institutional interventions that will address psychological distress and fear of COVID-19 during the pandemic, and provide psychotherapeutic support for students to be able to perform well at school. [50] however, found no relationship between psychological well-being and job performance.

*Psychological well-being of persons in rural and urban centers*. Previous studies [51, 52] have shown that there is a difference between the psychological well-being of people in rural and urban centers. For instance [52], examined the effect of mental health, well-being, and poverty in urban and rural communities in North Eastern Brazil, using purely quantitative methodology. A sample of 417 adult residents participated in the study. The authors found a significant difference in well-being and the prevalence of common mental disorders between residents of both communities, with a higher average well-being score in the rural context and a higher average score for the prevalence of common mental disorders in the urban sample.

[51] also examined the relationship between social exclusion and well- being among older adults in rural and urban areas in Barnsley, UK. Using the cross-sectional survey, a list of respondents classified into rural and urban households was created using the Barnsley Metropolitan Electoral Registration. A sample of 628 respondents from a rural location and 627 from an urban center were used. The authors found that the well-being of people residing in rural areas was relatively higher than the well-being of urban samples. According to the authors, the low level of well-being in urban areas was due to absence of neighborhood alienation.

Given the preceding review, the following hypotheses are formulated for this study;

1. *H1*: There is a statistically significant relationship between psychological well-being and job performance of health workers in rural areas.
2. *H2*: There is a statistically significant relationship between psychological well-being and job performance of health workers in urban areas.
3. *H3*: There is a statistically significant difference between psychological well-being and job performance of health workers at rural and urban areas.

**Fig 1:**
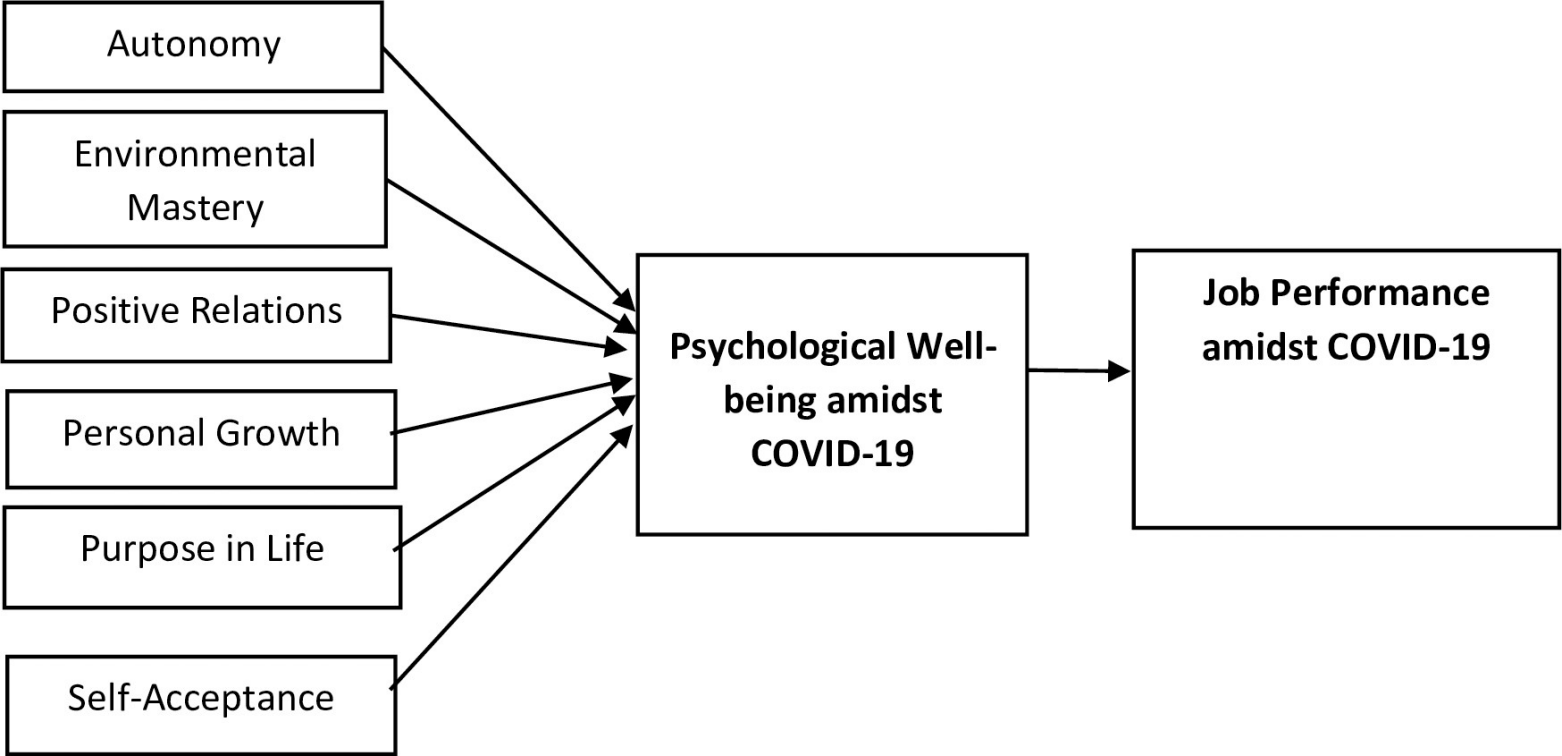
Conceptual framework.

The conceptual framework of this study is presented in Fig 1.

### Methods

#### Research design, population, sample size, and sampling procedure

The explanatory research design was used for this study. The target population consisted of general nurses, enrolled nurses, professional nurses, and midwives from the two hospitals in the Central Region of Ghana. In all, the target population was 800 nurses and midwives. Using [53] sample size determination table, this population corresponded with a minimum sample of 260. However, the researchers retrieved 262 questionnaires from the two hospitals. Thus, the sample used for this study is highly representative of the total population. The population was stratified into two groups; rural (Hospital A) and urban (Hospital B) as in Table 1.

**Table 1:** Number of respondents per hospital.

Name of hospital	Number of respondents	Percentage
Hospital A	119	45.5%
Hospital B	143	54.5%
Total	262	100.0%

#### Data collection instrument

A structured questionnaire was used for data collection for this study. According to [54], the structured questionnaire is the most appropriate data collection instrument for explanatory research. The 18-item version of Ryff Psychological Well-being Scale [55] was adapted for measuring psychological well-being in this study. Again, the 10 item-scale from by [56], often known as the Individual Work Performance Questionnaire was used to measure job performance. All items were measured on a Five-Point Likert Scale ranging from *‘1’- ‘Strongly Disagree’ to ‘5’- ‘Strongly Agree’*. This study was reviewed and approved by the Ethical Review Committees of both urban and rural hospitals. Also, consent was taken from participants before they were engaged in the study.

#### Data processing and analysis

The data processing was done with SPSS version 26 while SMART PLS 4 was used to analyse the processed data. In order to enhance the measurement model, some indicators with outer loadings of less than 0.7 (not statistically significant) were deleted as a choice rule. Because PLS-SEM validates measurement models before validating structural models, the evaluation of the models started with the measurement model [57]. The Cronbach’s Alpha (≥ 0.7) and Composite Reliability (≥ 0.7) were also calculated. All the Cronbach’s Alpha values were higher than the threshold of 0.7 [58]. The study assessed the dependability of the scale using the rho A statistic, with a threshold of ≥ 0.7. Convergent validity was evaluated using the Average Variance Extracted (AVE), which should ideally be at or above 0.5 for accurate quantification. Discriminant validity, gauging the uniqueness of each concept within a model, was discussed alongside the investigation and reporting of collinearity statistics to address biases and errors inherent in reflective models. Common method bias was also scrutinized with a VIF threshold of less than 3.3 indicating freedom from bias, although [59, 60] proposed a higher threshold of 5. Path loadings above 0.70 were considered typical for a reflective model, while effect size (f^2^) was utilized to measure the predictors’ contributions to changes in the dependent variable, with values above 0.35, between 0.15 and 0.35, and between 0.02 and 0.15 classified as strong, moderate, and weak, respectively. R-squared was employed as the primary effect size measure, with suggested thresholds for substantial, moderate, and weak effects. Adjusted R-squared and the coefficient of determination were also analyzed. Finally, predictive relevance was assessed through Q^2^, with values above 0 indicating good predictive relevance.

## Study results

### Measurement model

The measurement model includes an assessment of the reliability and validity of the scales and data [61]. Construct reliability was measured with Cronbach’s alpha (CA) and rho A; indicators’ reliability was assessed with item loadings; and convergent validity was measured with average variance extracted (AVE). Finally, discriminant validity was assessed with heterotrait-monotrait (HTMT) ratio. Table 2 illustrates the evaluation criteria for the model.

**Table 2:** Indicator loadings, variance inflation factor, reliability, and convergent validity statistics.

Variables		Indicator Loadings		Outer VIF		CA	Rho A	CR	AVE
		Hospital A	Hospital B	Hospital A	Hospital B				
Autonomy (AU)	Hospital A					0.858	0.859	0.913	0.778
	Hospital B					0.909	0.910	0.943	0.847
AU1		0.881	0.911	2.149	2.857				
AU2		0.893	0.934	2.242	3.502				
AU3		0.872	0.915	2.073	2.940				
Environmental Mastery (EM)	Hospital A					0.854	0.854	0.912	0.775
EM1	Hospital B	0.889	0.836	2.311	1.844	0.848	0.857	0.908	0.767
EM2		0.866	0.887	1.921	2.152				
EM3		0.885	0.902	2.225	2.343				
Positive Relations	Hospital A					0.847	0.853	0.907	0.765
PR1	Hospital B	0.904	0.851	2.827	2.160	0.829	0.833	0.898	0.745
PR2		0.866	0.901	2.424	2.513				
PR3		0.854	0.837	1.705	1.620				
Personal Growth (PG)	Hospital A					0.820	0.827	0.893	0.735
PG1	Hospital B	0.831	0.849	1.867	1.956	0.812	0.812	0.889	0.727
PG2		0.900	0.884	2.261	2.233				
PG3		0.840	0.824	1.683	1.557				
Purpose in Life (PL)	Hospital A					0.860	0.862	0.915	0.781
	Hospital B					0.856	0.863	0.912	0.776
PL1		0.882	0.856	2.218	2.160				
PL2		0.867	0.912	2.011	2.721				
PL3		0.903	0.873	2.399	1.954				
Self-Acceptance (SA)	Hospital A					0.788	0.814	0.875	0.702
SA1	Hospital B	0.757	0.796	1.552	1.573	0.798	0.804	0.881	0.712
SA2		0.896	0.884	2.124	2.043				
SA3		0.854	0.849	1.697	1.729				
Job Performance (JP)	Hospital A					0.927	0.931	0.939	0.606
JP1	Hospital B	0.722	0.767	2.047	2.210	0.942	0.945	0.951	0.660
JP2		0.849	0.853	3.114	3.726				
JP3		0.767	0.820	2.453	3.089				
JP4		0.713	0.853	2.095	3.858				
JP5		0.815	0.770	2.757	2.472				
JP6		0.851	0.839	2.951	3.087				
JP7		0.726	0.854	2.316	3.678				
JP8		0.717	0.697	2.285	2.172				
JP9		0.809	0.799	2.498	2.661				
JP10		0.798	0.856	2.395	3.511				

As in Table 2, all measuring items loaded above the required threshold, except JP8 (JP8 = 0.693) which did not have any significant impact on the model’s validity for the samples. Consequently, the reliability of the indicators used in the study was confirmed. Construct reliability (CA and CR > 0.70) was also attained in line with [62]. Rho_A scores also met the minimum criteria of 0.70 as expressed by [56]. As a result, all the measures demonstrated adequate levels of dependability. The AVE scores for all the constructs in this study are above the threshold of 0.5 hence, the validity criteria for AVE estimations were met.

#### Discriminant validity (DV)

Discriminant Validity (DV) is mostly determined in PLS-SEM by the heterotrait- monotrait (HTMT) ratio because of its robustness and dependability compared to Fornell-Larcker Criterion [63, 64]. Table 3 shows discriminant validity of this study using HTMT.

**Table 3:** HTMT ratio.

	AU	EM	JP	PWB	PG	PR	PL
**HTMT Ratio for Hospital B model**							
**AU**	0.000						
**EM**	0.749						
**JP**	0.967	0.840					
**PWB**	0.977	0.876	0.960				
**PG**	0.805	0.654	0.801	0.977			
**PR**	0.826	0.622	0.804	0.955	0.983		
**PL**	0.805	0.793	0.848	0.962	0.774	0.732	
**SA**	0.676	0.918	0.804	0.733	0.501	0.516	0.677
**HTMT Ratio Hospital A model**							
**AU**	0.000						
**EM**	0.742						
**JP**	0.926	0.728					
**PWB**	0.960	0.935	0.897				
**PG**	0.823	0.820	0.801	0.923			
**PR**	0.832	0.653	0.810	0.938	0.975		
**PL**	0.777	0.927	0.819	0.989	0.872	0.743	
**SA**	0.729	0.920	0.717	0.858	0.756	0.553	0.947

HTMT was assessed using a threshold of 1.0 [65, 66]. As in Table 3, all values were significantly different from the numerical value of 1.00. There is therefore, a reasonable level of discriminant validity for all the models.

#### Common method bias

The possibility for common method bias was explored since multiple variables were measured. The factor-based PLS algorithm was utilized to test the inner VIF [67]. As per [68, 69], ideal inner VIF scores must be less than 5.00 in order to rule out multi-collinearity of latent variables. Table 4 presents the VIF results for this study.

**Table 4:** Inner VIF.

	Hospital A	Hospital B
**AU**	2.646	3.133
**EM**	3.410	3.136
**JP**	0.000	0.000
**PWB**	0.000	0.000
**PG**	4.314	3.222
**PR**	3.816	3.294
**PL**	4.284	2.628

It can be deduced from Table 4 that the highest inner VIF value for Hospital A model is 4.314 and for Hospital B model is 3.294, implying that the constructs used in this study are devoid of multi-collinearity as maintained by Hair et al. (2014).

### Structural model

In assessing the structural model for this study, the common method bias, VIFs, path coefficient (β), statistical significance (t-stats), coefficients of determination (R^2^), effect sizes (f^2^), and predictive relevance (Q^2^) were evaluated [57].

#### Coefficient of determination

The coefficient of determination (R^2^) scores represents the predictability of a given model. R^2^ results vary from 0 to 1, with greater values representing better effects of prediction ability [58]. According to [69], effect size (f^2^) scores of 0.02, 0.15, and 0.35 imply low, moderate, and high impacts, correspondingly. The coefficient of determination (R^2^) and Effect size (f^2^) results for Hospital A model and Hospital B model are presented in Table 5.

**Table 5:** Coefficient of determination and effect size (f^2^).

	Hospital A Model			Hospital B Model		
	R^2^	Adjusted R^2^	f^2^	R^2^	Adjusted R^2^	f^2^
**PWB >JP**	0.722	0.719	2.592	0.831	0.830	4.910

As in Table 5, 72.2% of variations in job performance is predicted by psychological well-being of Hospital A samples, and 83.1% of variations in job performance is predicted by psychological well-being of Hospital B samples. The f^2^ scores indicate strong effects of psychological well-being on job performance (as per Cohen’s f^2^).

#### Path coefficients

**Fig 2:**
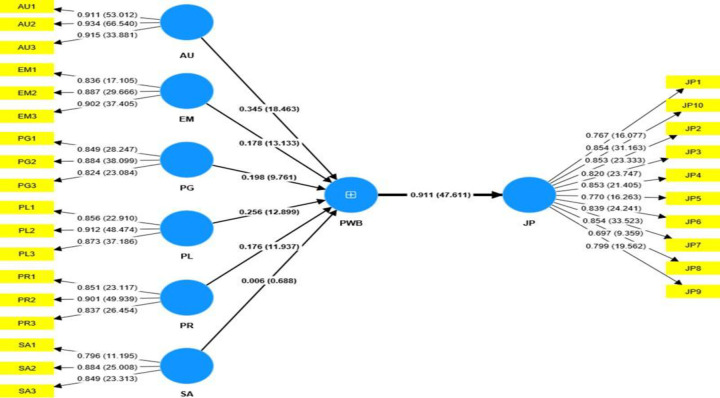
Outer loadings, path coefficients and bootstrapping results on the effect of psychological well-being (PWB) on job performance in urban hospitals (Hospital B).

**Fig 3:**
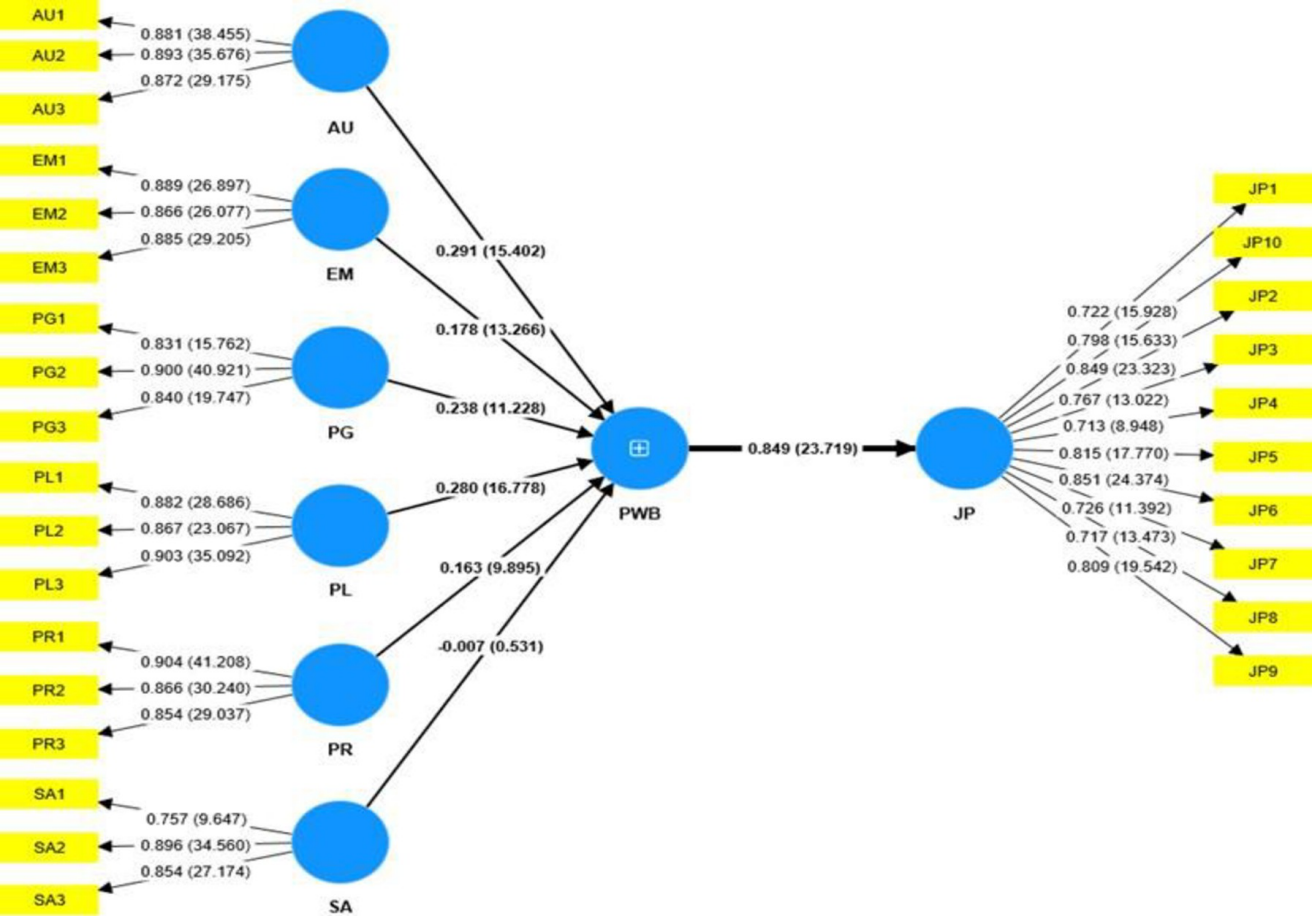
Outer loadings, path coefficients and bootstrapping results on the effect of psychological well-being (PWB) on job performance (JP) in rural hospitals (Hospital A).

Table 6 and Figs 2 and 3 illustrate the path coefficient for the model. According to [56], t-stats values above 1.96 correspond to p-values < 0.05 which represent statistical significance and vice versa. Generally, there was a statistically significant relationship between psychological well-being (PWB) and job performance (JP) for both the urban hospital, Hospital B (β = 0.911, t-stat = 47.611 and p-value = 0.000 < 0.005) as well as the rural hospital, Hospital A (β = 0.849, t-stat = 23.719 and p-value = 0.000 < 0.005).

**Table 6:** Path coefficients.

	Hospital B Model				Hospital A Model			
**PWB -> JP**	0.911	0.019	47.611	0.000	0.849	0.036	23.719	0.000

#### Predictive relevance (Q^2^)

The predictive relevance (Q^2^) of job performance (dependent variable) in the SEM is presented in Table 7. Citing [64], Q^2^ values > 0 are recommended. [64] provided Q^2^ criteria as 0.02 ≤ Q^2^ < 0.15 (weak effect), 0.15 ≤ Q^2^ < 0.35 (moderate effect), and Q^2^ > 0.35 (strong effect).

**Table 7:** Constructs and indicators predictive relevance.

	Hospital B model			Hospital A model		
Latent Variable	Q^2^	RMSE	MAE	Q^2^	RMSE	MAE
Job Performance	0.832	0.420	0.320	0.714	0.557	0.391
Psychological well-being	0.996	0.061	0.046	0.996	0.065	0.046

As in Table 7, job performance is strongly explained by psychological well-being in both Hospital B and Hospital A models, with Q^2^ > 0.35. Psychological well-being is also strongly explained by the exogenous variables.

### Differences between the effect of psychological well-being on job performance of health workers at Hospital A and Hospital B

In order to determine whether there was a statistically significant difference between the effect of psychological well-being on job performance of health workers in rural and urban communities, a multi-group comparison for Hospital A and Hospital B was performed. The results are presented in Table 8.

**Table 8:** Multi-group comparison for Hospital A and Hospital B.

	*Bootstrap MGA*	*Permutation mean difference*	*Parametric test*	*Welch-Satterthwait test*
AU -> PWB	-0.039*	0.052**	-0.039*	-0.039*
EM -> PWB	-0.009	-0.065	-0.009	-0.009
**PWB -> JP**	**-0.079** *	**-0.008** *	**-0.079** *	**-0.079** *
PG -> PWB	0.010	-0.032	0.010	0.010
PR -> PWB	0.004	0.049	0.004	0.004
PL -> PWB	0.002	-0.054	0.002	0.002
SA -> PWB	-0.002	0.045	-0.002	-0.002

Notes

* Significant at 0.10

**significant at 0.05

As in Table 8, nurses and midwives in Hospital A outperformed those in Hospital B in terms of the impact of autonomy (-0.039) on psychological well-being significantly. Alternatively, nurses and midwives in Hospital B outperformed those in Hospital A in terms of the impact of personal growth (-0.010) on psychological well-being significantly. However, although there is difference in the impact of environmental mastery (-0.009) and self-acceptance (-0.002) on psychological well-being for nurses and midwives in both Hospital A and Hospital B, this difference is not statistically significant. In either case, nurses and midwives in Hospital A outperformed those in Hospital B. Again, although the nurses and midwives in Hospital B outperformed those in Hospital A in terms of the impact of positive relations (0.004) and purpose in life (0.002) on psychological well-being, this difference was not statistically significant.

### Discussion of results

The main objective of this study was to examine the influence of psychological well- being on job performance of nurses and midwives in rural and urban hospitals amidst COVID-19 in Ghana. In this study, participants from Hospital A belonged to a hospital in a rural community whereas participants from Hospital B worked in an urban community. The discussion of results is organized into three sections: (a) psychological well- being and job performance of nurses and midwives in the rural community, (b) psychological well-being and job performance of nurses and midwives in the urban community, and (c) differences between the effects of psychological well-being on the job performance of nurses and midwives in rural and urban communities amidst COVID-19.

***H1*: *Psychological well-being and job performance of nurses and midwives in rural communities amidst COVID-19 (Hospital A)***

The path coefficient, p values, and t-statistic were used to test this hypothesis. The results indicated a favorable relationship between psychological well-being and job performance of Hospital A workers (β = 0.849, t-stat = 23.719 and p-value = 0.000 < 0.005), hence providing statistically significant reason for failing to reject H1. By implication, psychological well-being has favourable influence on the job performance of nurses and midwives in Hospital A. This means that any improvement in the ability to sustain a reasonable quality of life, which allows a person to get the most out of their daily activities without suffering excessive exhaustion would contribute to a rise in performance of nurses and midwives in rural hospitals. Again, any increment in their capacity to engage in constructive and supportive relationship with others at the workplace would impact significantly on the rate at which Hospital A health workers successfully accomplish their duties.

The preceding results are similar to the findings of [44] who posited that psychological well-being alongside financial, social, and physical well-being enhances work-related performance during uncertain times such as those experienced during COVID- 19 pandemic. Additionally, the findings of this study align with the findings of [47] who claimed that improved psychological health is beneficial for improving employee performance in all organizational structures. The findings of the study however, contrast with the findings of [48] who reported that sustaining close connections during a pandemic is challenging and stressful for many health workers, especially those in rural areas, and this results in impaired communication which ultimately leads to an inability to properly carry out job-related tasks.

***H2*: *Psychological well-being and job performance of nurses and midwives in urban communities amidst COVID-19 (Hospital B)***

The results of path coefficient, p values, and t-statistic indicated a favorable relationship between psychological well-being and job performance of the urban health workers (β = 0.911, t-stat = 47.611 and p- value = 0.000 < 0.005). Consequently, we fail to reject H2. By implication, an increase in psychological well-being would contribute to an increase in the efficiency of Hospital B health workers. This can be related to the findings of [45] which stipulated that psychological well-being has a direct supportive relationship with job-related performance. The results are also similar to those of [48] who found that emotional health at work accounts for 23% of the variation in individual job performance. In contrast with the findings of this study however [50], asserted that positive psychological well-being has no substantial influence on job performance as it is not a primary factor of job success.

***H3*: *Difference between the effect of psychological well-being on job performance of health workers at both rural and urban communities***

Finally, the study sought to test the differences in the effect of psychological well-being on the job performance of the two hospitals. This was accomplished through PLS-Multi-Group Analysis (MGA) and the Welch-Satterthwait test [48]. From Table 8 above, the outcomes of the multi-group comparative tests agree quite closely in general. Although psychological well-being affected job performance in both hospitals, the Bootstrap MGA (- 0.079*), permutation test (-0.008*), parametric test (-0.079*), and Welch-Satterthwait test (- 0.079*) revealed significant differences at 10% significant level between the effect of PWB on JP in respect of the two Hospitals. The results show that the effect of psychological well-being on job performance in the urban hospital (Hospital B) was greater than the effect of psychological well-being on job performance in the rural hospital (Hospital A). Practically, the direct influence of psychological well-being on job performance is 91.1% in Hospital B whilst 84.9% in the Hospital A sample.

## Theoretical implications

This study has a strong theoretical implication for employee psychological well-being and job performance in the context of a developing country. The theoretical contribution rests on the study’s extension of knowledge and application of the Job Demands-Resources (JD-R) theory to the health sector in developing countries such as Ghana. By reviewing the original theory as proposed by [17], and applying the assumptions to an empirical situation in Ghana, this study is expected to increase reader’s understanding of the Job Demands-Resources (JD-R) theory and how it is applied in real-life situations. Finally, by investigating the effect of psychological well-being on job performance of nurses and midwives in both rural and urban communities in Ghana based on the Job Demands- Resources (JD-R) theory, this study has succeeded in contributing to the existing body of knowledge on both variables and how they link to enhance the work of management.

## Practical implications

The current study has some important practical implications. First, the application of the Job Demands-Resources (JD-R) theory to explain the relationships between psychological well-being and employee job performance serves as a guide to management of the health sector selecting appropriate policy interventions on how employee performance may be enhanced through improvements in their psychological well-being. In other words, the current study will help managers to understand the importance of employees’ psychological well-being for work-related attitudes and behaviors. Second, the current study will help managers in the health sector to know the effect of a pandemic, especially COVID-19, on the job performance of health workers. As said earlier, at the core of COVID-19 lies a great number of psychological stressors such as, feelings of helplessness, fear of contracting the deadly coronavirus, the loss of fundamental sense of security, and worries about financial pressure which could potentially have an adverse effect on employee job performance [1, 5]. Finally, the findings from this study show that there are different aspects of job performance and various dimensions of psychological well-being, and that any attempt to promote a particular type of job performance can adversely affect the others. The same applies to an attempt to promote any particular type of well-being in a given situation. Thus, following the findings from this study, management is required to assess the trade-off between the different components of psychological well-being and job performance before adopting any policy intervention.

## Conclusion and suggestions for future research

This study proposed a model to understand the relationship between psychological well-being and job performance of nurses and midwives in both rural and urban communities in the Central Region of Ghana. The results revealed that despite the fears and anxieties associated with COVID-19 pandemic, nurses and midwives at both Hospital A and Hospital B maintained exceptional levels of psychological well-being (hedonic and eudaimonic) and job performance. This may be due to the fact that management of both hospitals have high interests in the psychological well-being of their workers, and have instituted measures that enhance the workers’ psychological well-being. The current study focused on hospitals in the Central Region of Ghana. Further studies could be conducted to include more hospitals in different regions for a cross-regional analysis.

## Supporting information

S1 File (PDF)
