# Supplementary material for: Psychological well-being and job performance of nurses and midwives amidst COVID-19 in Ghana; a multi-group analysis
Source: PLoS One. 2024 Aug 28;19(8):e0303855. doi: 10.1371/journal.pone.0303855 (PMC11356392; doi:10.1371/journal.pone.0303855)
Supplement: S1 File — (PDF) [file pone.0303855.s001.pdf]

*In case of reply the reference number
and the date of this
Letter should be quoted*

Our Ref.: CCTH

Your Ref.:

P. O. Box CT.1363
Cape Coast
CC-071-9967
Tel: 03321-34010-14
Fax: 03321-34016
Website:
www.ccthghana.org
email:
info@ccthghana.com

12th July, 2022

Nester Kumiwaa Owusu
Department of Human Resource Management
School of Business
University of Cape Coast
Cape Coast

Dear Madam,

ETHICAL CLEARANCE – REF: CCTHERC/EC/2022/118

The Cape Coast Teaching Hospital Ethical Review Committee (CCTHERC) has reviewed your research protocol titled, **"Psychological Wellbeing and Job Performance of Health Workers Amidst COVID-19; Evidence from Selected Hospitals in Central Region"** which was submitted for ethical clearance. The ERC is glad to inform you that you have been granted provisional approval for implementation of your research protocol.

The CCTHERC requires that you submit periodic review of the protocol and a final full review to the ERC on completion of the research. The CCTHERC may observe or cause to be observed procedures and records of the research during and after implementation.

Please note that any modification of the project must be submitted to the CCTHERC for review and approval before its implementation.

You are required to report all serious adverse events related to this study to the CCTHERC within ten (10) days in writing. Also note that you are to submit a copy of your final report to the CCTHERC office.

Always quote the protocol identification number in all future correspondence with us in relation to this protocol.

Yours sincerely,

Dr. Stephen Laryea
Medical Director
For: Prof. Ganiyu Rahman, Chairman ERC
